# Internal valence modulates the speed of object recognition

**DOI:** 10.1038/s41598-017-00385-4

**Published:** 2017-03-23

**Authors:** Matthew F. Panichello, Kestutis Kveraga, Maximilien Chaumon, Moshe Bar, Lisa Feldman Barrett

**Affiliations:** 1 Princeton Neuroscience Institute, Princeton, NJ USA; 2 Athinoula A. Martinos Center for Biomedical Imaging, Massachusetts General Hospital & Harvard Medical School, Charlestown, MA USA; 3 Berlin School of Mind and Brain, Humboldt-Universität zu Berlin, Berlin, Germany; 4 Gonda Multidisciplinary Brain Research Center, Bar-Ilan University, Ramat-Gan, Israel; 5 Interdisciplinary Affective Science Laboratory, Department of Psychology, Northeastern University, Boston, MA 02115 USA; 6 Department of Psychiatry, Massachusetts General Hospital & Harvard Medical School, Charlestown, MA USA

## Abstract

Brain regions that process affect are strongly connected with visual regions, but the functional consequences of this structural organization have been relatively unexplored. How does the momentary affect of an observer influence perception? We induced either pleasant or unpleasant affect in participants and then recorded their neural activity using magnetoencephalography while they completed an object recognition task. We hypothesized, and found, that affect influenced the speed of object recognition by modulating the speed and amplitude of evoked responses in occipitotemporal cortex and regions important for representing affect. Furthermore, affect modulated functional interactions between affective and perceptual regions early during perceptual processing. These findings indicate that affect can serve as an important contextual influence on object recognition processes.

## Introduction

Perception is a proactive process influenced by the state and history of the perceiver. This includes the perceiver’s affect, such as his or her subjective feelings of valence (pleasure/displeasure) and arousal. Affect influences both the process and contents of perception^1–7^. Indeed, evidence suggests that valence may modulate the speed and sensitivity of object recognition. Observers induced into a neutral state require longer stimulus presentation times to identify masked neutral words than do observers induced into a negative state, while observers experiencing positive affect require an even longer presentation to achieve the same performance^8^. Additionally, observers are better able to discriminate the orientation of low-contrast gratings when primed with negative rather than neutral faces^9^. These behavioral findings provide evidence suggesting that negative affect facilitates and positive affect hinders perceptual processing.

The neural mechanisms that allow valence to modulate perception are not well understood. Anatomical and brain imaging studies, however, both point to strong connections between the brain’s affective system and visual system. Via these connections, a perceiver’s momentary affect can influence perception. A network of cortical and subcortical structures process affect^10^. Orbitofrontal cortex (OFC), a region containing key components of affective circuitry, is particularly well positioned to influence perceptual processing^11, 12^. OFC projects directly to regions in the ventral visual stream known to be critical for visual perception^13–19^. Furthermore, OFC is strongly and reciprocally connected to the amygdala, which sends excitatory projections as far back as primary visual cortex^20, 21^. Thus, a state change in affective circuitry (e.g., following affect induction) could lead to an increase in excitability in visual regions via top-down modulation, thereby facilitating the rapid propagation of visual information^22^. Consistent with an increase in the excitability of visual regions, negative affect has been shown to decrease oscillatory signatures of inhibitory activity in occipital EEG electrodes^8^.

In the present study, we tested the hypothesis that valence would modulate the speed of object recognition, and that this speeded perception would be mediated by faster evoked responses in visual cortex and in affective circuitry positioned to provide top-down control of perception. Participants who began in a neutral state were induced into a negative or positive state and then performed an object identification task while their brain activity was recorded using magnetoencephalography. MEG has good spatial resolution and exceptional temporal resolution, allowing us to examine both the magnitude and latency of region-specific evoked activity. To our knowledge, this is the first time these techniques have been used to study affective modulation of object perception. Consistent with our hypothesis that valence modulates the speed of object recognition, we predicted that participants in a negative and positive state would differ in response times during the object recognition task. Furthermore, we predicted that this difference in response time would correspond to differences in the latency of stimulus-evoked responses at the neural level.

## Results

### Confirmation of affect manipulation

Each participant was randomly assigned to receive either positive or negative affect induction. To ensure a robust and consistent manipulation of affect, induction was applied repeatedly over the course of the experiment. Specifically, the experiment consisted of alternating affect induction periods, during which participants were presented with affectively-charged images and music, and object recognition periods, during which participants completed the object recognition task (see methods). Affect induction stimuli had been previously normed and were selected to differ in valence but not arousal.

**Figure 1:**
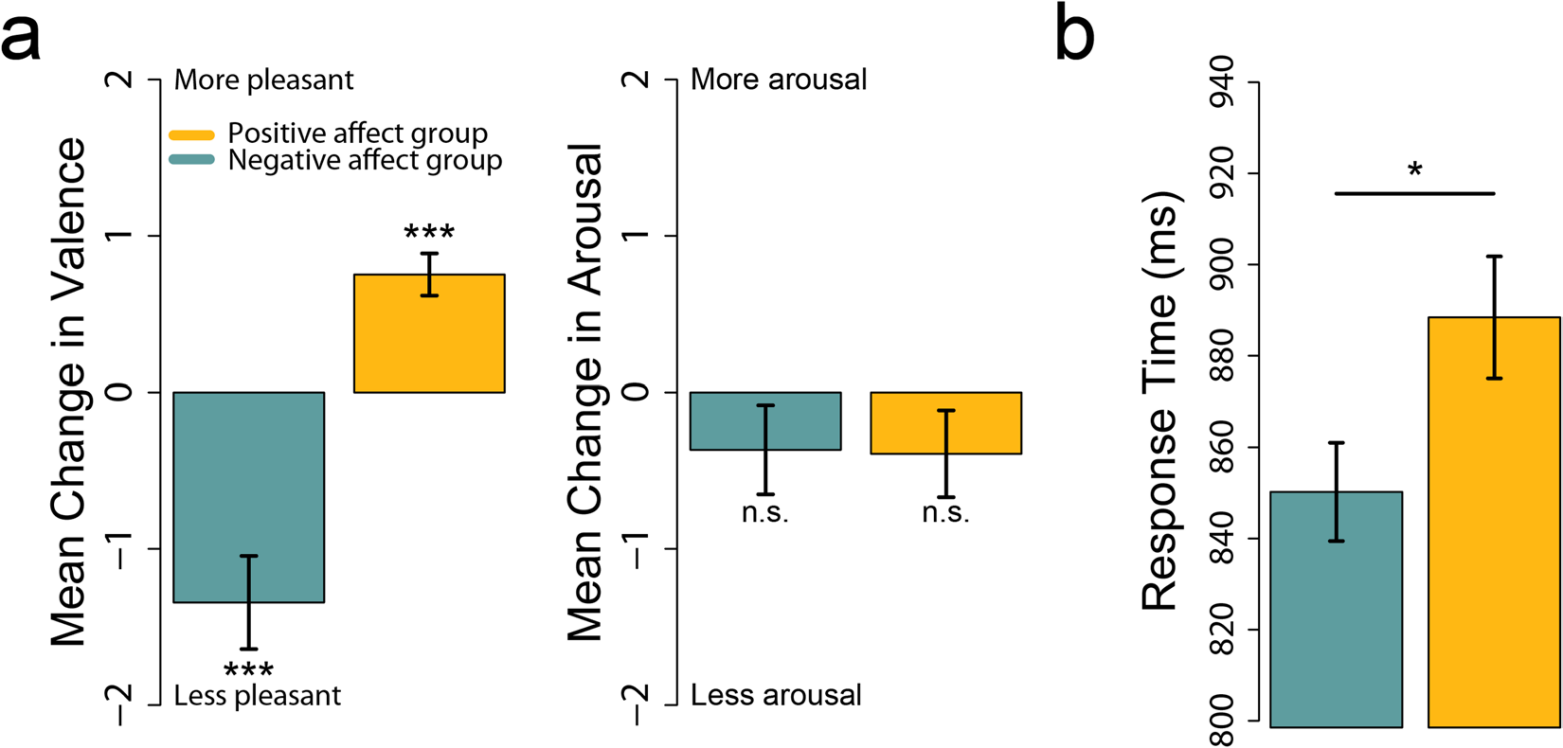
Confirmation of affect induction, and influence of affect on response time. (**a**) Affect induction periods induced a change in self-reported valence, but not arousal. (**b**) During the object recognition task, the negative affect group displayed faster response times than the positive affect group. Error bars indicate standard error of the mean.

Immediately before and after each affect induction period, participants rated their subjective valence on a 0–10 scale, where 0 indicated a highly unpleasant and 10 a highly pleasant state. Participants also rated their arousal on a similar scale, where 0 indicated extremely low arousal and 10 indicated high arousal. Differences between these between pre- and post-induction ratings confirmed that the affect induction procedure reliably altered participants’ valence but not arousal (Fig. 1a). Specifically, we subtracted each participant’s valence and arousal ratings before each affect induction period from their valence and arousal rating immediately after each affect induction period, and then averaged these difference scores. Positive affect induction periods elicited an increase in positive valence (t(9) = 5.57, p-value < 0.001) while negative affect induction periods elicited an increase in negative valence (t(9) = −4.50, p = 0.001). In contrast, positive affect induction (t(9) = −1.41, p = 0.192) and negative affect induction (t(9) = −1.29, p = 0.229) did not alter arousal, as intended. These ratings confirm that the induction periods consistently evoked different affect in the two groups of participants over the course of the experiment.

We also measured participant’s affect immediately before and after the entire experiment using the Positive Activation and Negative Activation Schedule (PANAS). Analysis of the PANAS scores revealed that individuals within the positive and negative affect groups reported similar valence before the first affect induction block (T1), but they differed substantially by the end of the experiment (T2). A 2 (affect: positive & negative) × 2 (timepoint: T1 & T2) repeated measures ANOVA revealed a main effect of affect induction F(1,17) = 14.83, p = 0.001 and timepoint F(1,17) = 21.42, p < 0.001, qualified by an affect × timepoint interaction F(1,17) = 5.25, p = 0.035. Post hoc t-tests revealed both groups reported more negative valence after the task. Critically, however, the negative affect induction group reported a more dramatic increase in negativity and differed substantially from the positive induction group after the task.

### Effect of valence on behavior and neural responses: between subjects

#### Response time and accuracy

On each trial of the object recognition task, participants were presented with a line drawing of an everyday object and made a speeded response indicating whether or not the object would fit in a shoebox. Consistent with our predictions, response times on the object recognition task differed between the two affect conditions. Participants who were induced into a negative state were faster than those in a positive state (Fig. 1b, t(18) = 2.22, p = 0.039).

Although it was not of primary interest, we also examined response accuracy for object recognition responses. Affect did not influence accuracy (mean negative affect group accuracy: 83.0%, mean positive affect group accuracy: 82.9%, t(18) = −0.05, p = 0.958).

#### Latency and amplitude of evoked responses

**Figure 2:**
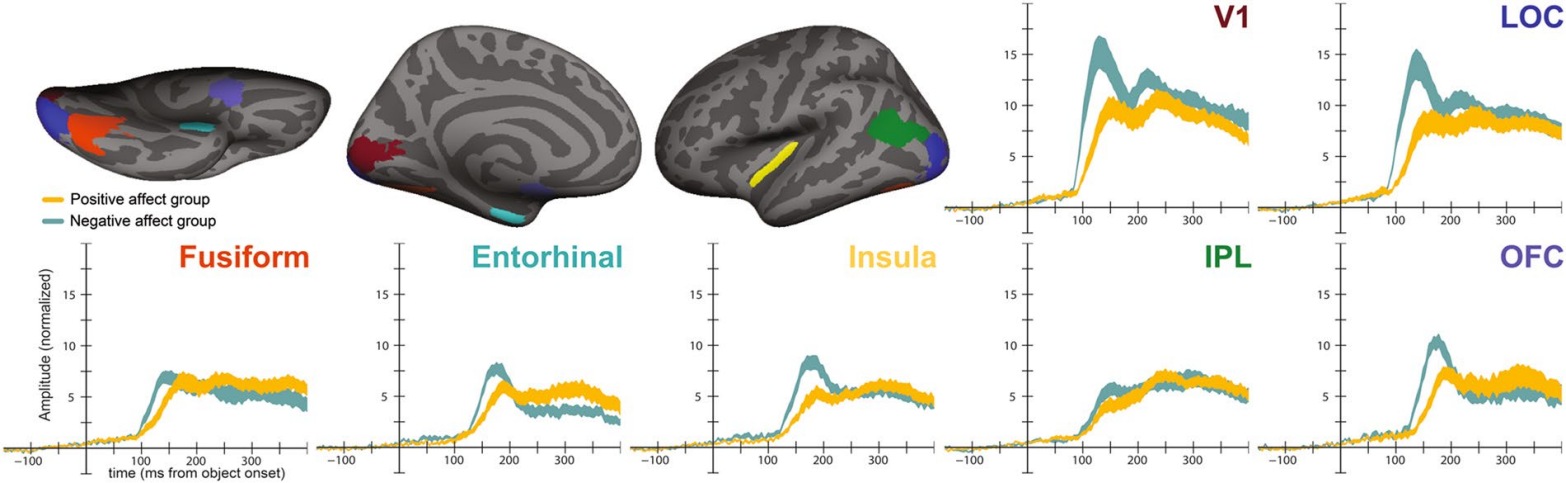
Regions of interest and average stimulus-evoked responses. Top-left: ventral, medial, and lateral views of the left hemisphere of an inflated brain showing a representative set of ROI labels. Note that we generated anatomically-constrained, functionally-defined ROIs for each individual participant. Surround: mean stimulus-evoked responses extracted from each region of interest and averaged across hemispheres (with SEM range shaded). Maroon = V1, blue = lateral occipital complex, orange = fusiform cortex, teal = entorhinal cortex, yellow = insula, green = inferior parietal lobule, purple = OFC. Shaded region indicates standard error of the mean.

We next asked if valence modulated the latency and amplitude of evoked responses across a range of ROIs selected a priori for their involvement in visual perception, affective processing, and object recognition (Fig. 2; Supplementary Figures 1 and 2). Evoked response latency was assessed using two statistics: onset latency, or time from stimulus onset to the point when the evoked response began to rise above baseline; and peak latency, time from stimulus onset to the maximum value of the evoked response. We examined these two statistics because we reasoned that both earlier onset of evoked activity or reduced time to peak response could be markers of facilitation with consequences for behavior. All evoked statistics were analyzed using a 3-way ANOVA with factors affect induction condition (negative or positive), ROI, and hemisphere.

**Figure 3:**
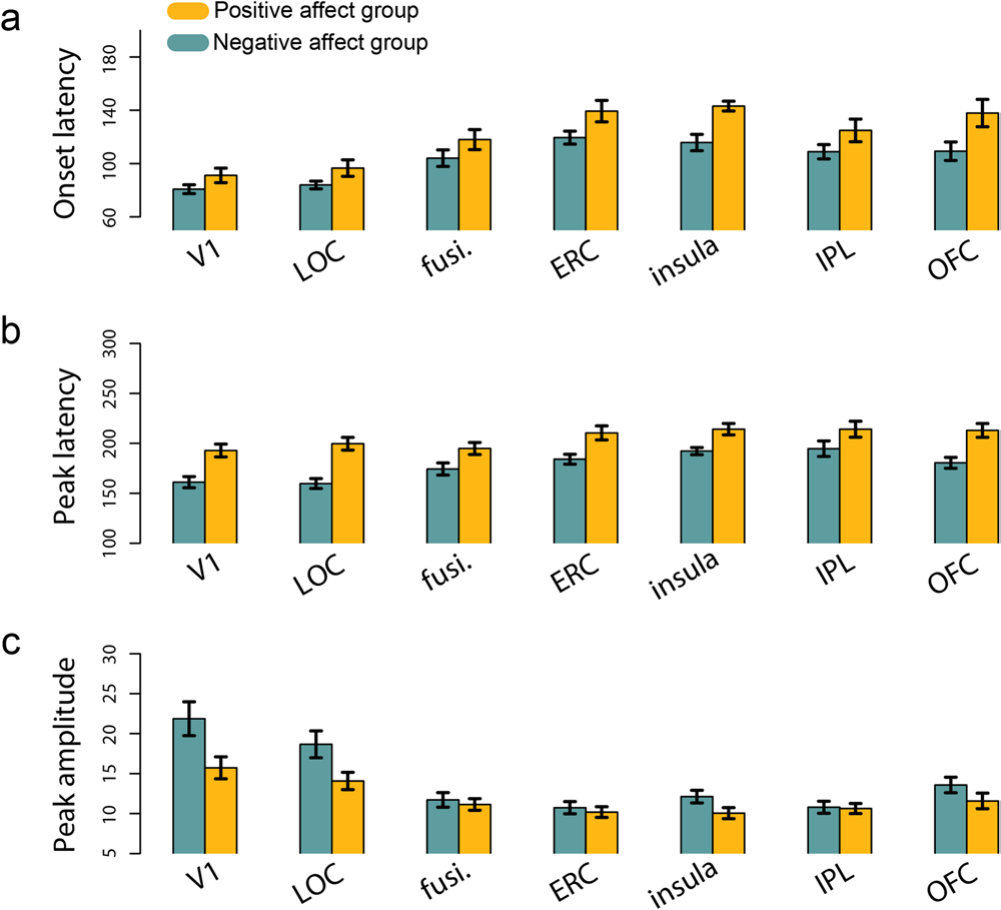
Differences in evoked response latency and amplitude between affect groups. (**a**) Mean onset latency (**b**) mean peak latency and (**c**) mean amplitude across regions of interest, broken down by negative and positive affect group. LOC = lateral occipital complex, fusi. = fusiform cortex, ERC = entorhinal cortex, IPL = inferior parietal lobule, OFC = orbitofrontal cortex. Error bars indicate standard error of the mean.

We found a main effect of affect condition on onset latency (F(1,18) = 8.21, p = 0.010). Across ROIs, negative affect was associated with faster onset of evoked responses (Fig. 3a). There was also a main effect of ROI (F(6,234) = 32.32, p < 0.001). There was no effect of hemisphere, nor any interactions among factors.

Affect condition also modulated the *peak* latency of evoked responses (F(1,18) = 31.91, p < 0.001). Again, negative affect was associated with faster neural responses (Fig. 3b). There was also a main effect of ROI (F(6,234) = 9.05, p < 0.001). There was no main effect of hemisphere, nor any interaction among factors.

In addition to the timing of cortical responses to the object stimuli, affect influenced the amplitude of these responses (F(1,18) = 9.94, p = 0.006). There was also a main effect of ROI (F(6,234) = 29.34, p < 0.001) and an affect × ROI interaction (F(6,234) = 3.61, p = 0.002). To further investigate this interaction, we carried out post-hoc t-tests comparing the amplitude of responses for the two affect groups in each ROI, collapsing across hemisphere. Individuals in the negative affect condition had greater response amplitudes in V1 and LOC than individuals in the positive affect condition (t(18) = 2.43 & 2.29, p = 0.026 & 0.034 uncorrected, Fig. 3c).

Collectively, these results suggest that individuals induced into an unpleasant state tended to perform the object recognition task more quickly, and also tended to display faster (and, in some regions, larger) evoked responses throughout visual and affective regions.

### Effect of valence on behavior and neural responses: within subjects

#### Response time and accuracy

Having observed differences in behavioral and neural markers of object recognition between our affect groups, we next asked if spontaneous fluctuations in valence *within* subjects also predicted similar changes in behavior and neural responses. Indeed, over the duration of the task, fluctuations in an individual’s self-reported valence predicted his or her response time. Every twenty-five trials, participants rated their valence and arousal on a scale of 0–10 and then the affect induction procedure was re-administered. We modeled trial-by-trial response time as function of valence rating as well as control variables including arousal rating, trial number, block number, image characteristics, and induction group. The model also accounted for random effects in the model intercept due to inter-subject variability. Response time increased with the positivity of valence (i.e. subjective pleasantness, b = 11.03, p < 0.001) but did not systematically vary with arousal (b = −1.19, p = 0.496). Thus, within subjects, a more negative state was associated with faster response times.

#### Latency and amplitude of evoked responses

We next asked if response latency and amplitude varied with self-reported fluctuations in valence (again controlling for arousal and other factors), using a Bonferroni adjusted alpha of 0.0036 to correct for tests across 7 ROIs and 2 hemispheres. Mixed effects modeling revealed a significant relationship between valence and the amplitude of evoked responses in left V1 (b = −23.32, p < 0.001) and right V1 (b = −22.09, p < 0.001). There was no relationship between valence and latency in any ROI.

#### Relating valence, neural responses, and behavior

Within subjects, we observed that when individuals reported less pleasant valence they were faster at the object recognition task and also displayed larger evoked responses in left and right V1. We next asked if the influence of valence on response time was mediated by the amplitude of these early visual responses. Briefly, amplitude in each region was said to mediate the effect of valence on response time if valence tended to successfully predict amplitude and if amplitude tended to successfully predict response time even when controlling for valence (see Materials and Methods). Mediation analyses revealed that as subjective pleasantness decreased, trial-by-trial amplitude of the evoked response in left V1 increased, which in turn was associated with faster response times (Z_ab_ = 2.93, p = 0.021).

### Effect of valence on OFC-ventral stream interactions during perception

Having demonstrated that valence modulates the latency and amplitude of evoked responses within particular brain regions, we next asked if valence modulated functional interactions between brain regions. Functional interactions between OFC and fusiform cortex have previously been associated with faster and more accurate object recognition using both MEG^23^ and fMRI^24^. Given the role of OFC in representing valence, and the fact that we observe a behavioral advantage during object recognition for negative valence, we hypothesized that valence would modulate the strength of functional interactions between OFC and fusiform cortex.

To test this hypothesis, we used the current estimates extracted from each region of interest to characterize cross-cortical communication between these ROIs. Cortical regions engaged in the exchange of information display synchronized oscillatory dynamics^25^. Taking advantage of the temporal resolution of MEG, we can measure the degree of synchronization in these oscillatory dynamics across brain regions and quantify this as a phase-locking factor (PLV; see methods). Thus, an increase in PLV values between two brain regions is a marker of an increase in functional connectivity^26^.

**Figure 4:**
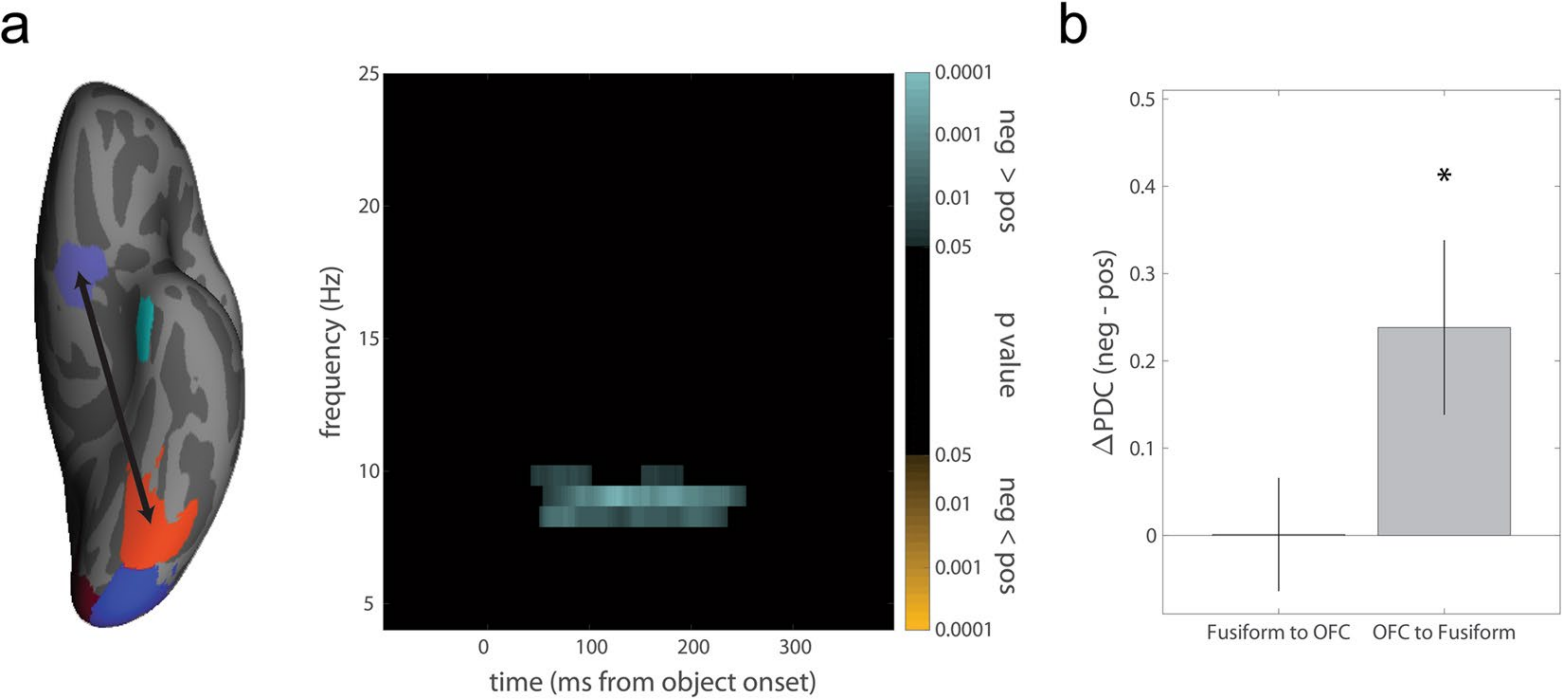
Functional connectivity varies with fluctuations in valence within subjects. (**a**) Left: There was significantly greater phase-locking between left OFC and fusiform cortex on trials in which subjects were experiencing more negative valence (median split). Right: The color-map p values represent the results of paired-samples t-tests between phase-locking values derived from the median split of trials. The correction for multiple comparisons was performed by using a nonparametric cluster permutation test described in *Methods*; only clusters surviving this correction are shown. When subjects were in a relatively negative state, we observe significantly greater phase-locking in the α-band shortly after stimulus onset. (**b**) Difference in partial directed coherence from fusiform to OFC and from OFC to fusiform cortex on trials when subjects were experiencing relatively negative or positive valence (median split).

To assess changes in phase-locking within subjects, we performed a median split of each subject’s correct trials based on their self-reported valence. There was a significant increase in phase-locking (p = 0.026; all MEG phase-locking p values corrected for multiple time frequency point comparisons as described in methods) between left OFC and fusiform cortex (Fig. 4a). This increase in phase-locking began ~50 ms after stimulus onset, sufficiently early to influence the dynamics of object recognition, and was observed in the α-band, consistent with previous findings^23^. There were no differences in OFC-fusiform phase-locking between subjects.

As an exploratory analysis, we also looked for modulation of phase-locking between OFC and our other ventral visual ROIs (LOC, ERC, and V1). Within subjects, there was a relatively late β-band increase in synchrony between left OFC and LOC (p = 0.059, Supplementary Figure 3a) on trials during which participants reported more negative valence. Between subjects, there was greater α- and low β-band phase-locking between left OFC and ERC for individuals in the negative affect group (p = 0.049, Supplementary Figure 3b).

These phase-locking analyses demonstrate the presence of an interaction between OFC and ventral visual regions, but cannot resolve the directionality of this interaction. How does valence modulate the feedforward and feedback flow of information between OFC and these regions? We employed partial directed coherence (PDC), a measure related to granger causality, to characterize the information transfer to and from OFC. Overall, there was significant transfer of information both from left fusiform cortex to OFC (PDC = 4.91, p < 0.001, randomization test) and from left OFC to fusiform cortex (PDC = 4.69, p < 0.001), consistent with the recurrent nature of cortical interactions. To assess how valence modulated these interactions, we again performed a median split of trials for each subject based on self-reported valence and recomputed the PDC in each direction for each half of the data. Negative valence was associated with an increase in information flow from OFC to fusiform cortex (Fig. 4b, t(18) = 2.45, p = 0.025) but not from fusiform cortex to OFC (t(18) = 0.02, p = 0.987). Directly comparing the two PDC difference scores, there was a greater increase in information flow from OFC to fusiform as compared to fusiform to OFC when subjects were in a relatively negative state (t(18) = 2.25, p = 0.036).

Additionally, there was significant bidirectional transfer of information between left LOC and OFC (PDC = 4.73 and 4.53, both p < 0.001) and between left ERC and OFC (PDC = 6.22 and 5.77, both p < 0.001). On trials during which subjects reported relatively more negative valence, there was an increase in information flow from OFC to LOC (t(18) = 2.90, p = 0.010) but not from LOC to OFC (t(18) = 1.61, p = 0.124, Supplementary Figure 3c). However, these two difference scores did not significantly differ from each other (t(18) = 0.81, p = 0.431). There were no differences in PDC scores from ERC to OFC (t(18) = −0.77, p = 0.451) or OFC to ERC (t(18) = −0.18, p = 0.857) between the two affect groups (Supplementary Figure 3d).

## Discussion

Our study provides evidence that the affect of a perceiver influences the dynamics of object perception. Affect modulates the latency and amplitude of evoked responses throughout affective and visual regions. Affect induction did not elicit changes in participant arousal, and within-subjects analyses revealed an effect of valence on response time and neural responses, even when arousal ratings made by the participants during the task were included as a covariate. Thus, differences in arousal are unlikely to account for these results. These findings lend support to the growing body of findings that negative affect facilitates visual perception^8^, perhaps by inducing a neural state that supports rapid propagation of visual information throughout the brain.

Our affect manipulation provides strong evidence that the valence of an observer can modulate the speed of behavioral and neural markers of object recognition. Based on this manipulation, however, one cannot specifically conclude whether negative affect facilitates perceptual processing, positive affect hinders perceptual processing, or both. Also, interpretation of our findings is limited by sample size (10 subjects per affect induction group). To address these issues, we examined how fluctuations in valence impacted behavior and neural responses within subjects. Regardless of whether subjects had been pushed into a negative or positive valence regime by our affect induction procedure, response times increased with smaller, spontaneous increases in valence. Furthermore, within subjects, we replicated influences of valence on response amplitude in V1. Regardless of whether subjects were in a positive or negative state overall, an increase in negative valence increased the amplitude of evoked responses. These results suggest that negative affect facilitates *and* positive affect hinders perceptual processing.

In addition to modulating the latency and amplitude of evoked responses, changes in affect also altered communication between cortical regions. Both within and between subjects, negative affect was associated with greater phase-locking between OFC and ventral visual regions. Furthermore, negative valence was selectively associated with increased flow of information from OFC to fusiform cortex and LOC, consistent with a modulatory influence of OFC on object recognition processes. OFC-ventral stream interactions have been previously been shown to be associated with successful object recognition^23, 24^, consistent with the behavioral advantage we observe for negative affect in the present study. Under typical conditions, top-down feedback from OFC to the ventral visual stream may facilitate perceptual processing by communicating predictions about objects in the environment^11, 27^. Future research should investigate whether negative affect enhances this particular form of top-down feedback, or facilitates perception via an additional mechanism that also relies on OFC-ventral stream interactions.

Affective experience plays a key role in assigning adaptive value to our environment; we recoil from unpleasant (threatening, unsanitary, unrewarding) things and are drawn to pleasant (beneficial, salutary, rewarding) ones. It is tempting to explain our results in an evolutionary framework in which negative affect, signaling a potentially threatening or dangerous environment, enhances perceptual processing to facilitate withdrawal from the situation.

## Methods

### Participants

Twenty right-handed volunteers with normal or corrected to normal vision completed the study (9 females, mean age = 27.5(4.4)). Participants were screened to ensure no history of mental illness or use of psychoactive medication. All participants gave informed, written consent, and were paid for taking part. The study was approved by the Massachusetts General Hospital Institutional Review Board and carried out in accordance with the approved guidelines.

### Stimuli

We created 500 line drawings of everyday objects by editing color photos in GIMP (the GIMP team). These images were altered on-line during the task in one of three ways: foreground and background were defined by either differences in color (red/green), differences in luminance (light/dark), or differences in both color and luminance. These manipulations were introduced to test an unrelated set of hypotheses that will be reported elsewhere. We collapse across these conditions in the present report.

During the main task, all images were displayed using the stimulus presentation package Psychtoolbox running in Matlab (Mathworks) on a Macintosh Macbook Pro. The stimuli (256 × 256 pixels) were projected onto a translucent screen positioned 140 cm in front of the seated participant and subtended approximately 5.7° of visual angle. The entire display was 42 cm × 55 cm in size with a resolution of 1,024 × 768 pixels and a refresh rate of 75 Hz using an LP350 DLP projector (InFocus).

### Task Design

Each participant was randomly assigned to receive either positive or negative affect induction (10 participants for each group). To ensure a robust and consistent manipulation of affect, induction was applied repeatedly over the course of the experiment. Specifically, the task consisted of alternating affect induction periods, during which participants experienced evocative images and music, and object perception periods, during which participants would complete 25 trials of the object recognition task. Affect induction and object perception periods are described in more detail below.

Each affect induction period consisted of a 75 sec slideshow of images and music with strong negative or positive valence. Images were drawn from the International Affective Picture System^28^ and were each presented for 7.5 seconds (for a total of 10 images per slideshow). Music consisted of segments from Albinoni’s “Adagio in G minor” (positive valence) and Holst’s “The Planets, Op. 32.2” (negative valence). Music was normed internally to ensure the pieces differed in valence but not arousal.

Object perception periods consisted of 25 trials of an object recognition task. On each trial, participants were presented with a line drawing of an object for 500, 550, or 600 ms. To index recognition, participants were asked to use a response keypad to indicate whether or not the object would “fit into a typical shoebox”. Participants could also indicate if they did not recognize the object. There was a jittered inter-trial interval ranging from 500 to 1,500 ms between trials, sampled from a uniform distribution.

Participants completed 20 object perception periods (for a total of 500 trials of the object recognition task), with each object perception period preceded by an affect induction period.

### Affect induction checks

We administered several questionnaires designed to assess the effectiveness of our affect induction procedure. Participants completed the brief Positive Activation and Negative Activation Schedule (PANAS^29^) to assess their affective experience twice during the study, once immediately before and once immediately after the sequence of affect induction and object perception periods constituting the main task. We obtained a positive activation (PA) and a negative activation (NA) score by taking the mean of the 10 items for each subscale. To compute a unitary measure, the PA subscale and the inverse of the NA subscale were summed and averaged to produce a single statistic for each participant at each time point, ranging from 0 (most negative) to 6 (most positive). One participant did not complete the second PANAS and was excluded from this particular analysis. Participants also rated their pleasure/displeasure (valence) and arousal on a scale from 0 to 10 before and after each affect induction block by marking a position within an on-screen affect grid^30^. Participants were familiarized with the concepts of valence and arousal before the experiment and told that ratings of 0, 5, and 10 corresponded to ‘very unpleasant’, ‘neutral’, and ‘very pleasant’ valence states and ‘very inactive/lethargic’, ‘neutral’, and ‘very active/excited’ arousal states.

### MEG acquisition

The magnetoencephalogram was recorded using a 306-channel (204 Planar gradiometers, 102 magnetometers) Neuromag Vectorview whole-head system (Elekta Neuromag Oy) housed in a three-layer magnetically shielded room (ImedcoAG). Participant head position was monitored using four head-position indicator (HPI) electrodes affixed to the participant’s head. HPI position as well as that of multiple points on the scalp were recorded with a magnetic digitizer (Polhemus FastTrack 3D) in a head coordinate frame defined by anatomical landmarks. Eye blinks were monitored with 4 electrooculogram (EOG) sensors positioned above and beside the participants’ eyes. MEG, EOG, and HPI data were sampled at 600 Hz, bandpass filtered from 0.1–200 Hz, and stored for offline analysis. MEG data were analyzed using the MNE analysis package^31^ and custom scripts.

### Data preprocessing and averaging

After recording, the raw data were lowpass filtered at 120 Hz and signal-space projection was applied to the data to remove cardiac and ocular artifacts^32, 33^. MEG data time-locked to the onset of the object stimuli (300 ms prestimulus to 400 ms poststimulus) were extracted and data from individual trials were excluded from further analysis if they met any of the following criteria: (1) amplitude of any gradiometer exceeded 2,000 fT/cm; (2) amplitude of any magnetometer exceeded 4000 fT; (3) EOG amplitude exceeded 150 µV; (4) the participant made an incorrect judgment of object size on that trial; or (5) the participant’s response time on the trial did not fall within 2 standard deviations of his or her mean response time.

A high-resolution structural MRI, acquired on a Siemens Allegra 3 T scanner (Siemens Medical Solutions), was used to construct a forward model and visualize MEG sources. All participants had participated in an MRI experiment at MGH in the past and consented to share their MRI data for this purpose. A single-layer boundary element model was constructed from the anatomical MRI, and it was used as a forward model to constrain MEG source localization to the vertices of a triangular mesh model of cortex. The distribution of currents across vertices at each time point was estimated using the minimum-norm estimate^31^. These current estimates were transformed into spatiotemporal maps of cortical activation over time (dynamic statistical parametric maps or dSPMs). dSPMs are a dimensionless statistical test variable computed by dividing the estimated current amplitude at a particular point in cortex by the estimated variance at that point during a pre-stimulus baseline period, providing a noise-normalized representation of cortical activity over time^34^.

### ROI selection

Regions of interest (ROIs) were selected based on their relevance to early visual processing: primary visual cortex (V1); object recognition: lateral occipital complex^35^ (LOC), fusiform gyrus^36^, entorhinal cortex^37^ (ERC); affective processing: insula^38, 39^, orbitofrontal cortex^40^ (OFC); and top-down facilitation of object recognition: OFC, perhaps by way of IPL^23, 24, 27, 41^. We did not attempt to define an ROI for the amygdala because the MEG signal is primarily generated by cortical pyramidal cells^42^.

Subject-specific anatomically-constrained functional ROIs were created for each participant using the following procedure. Anatomical parcellation of each participant’s anatomical data was performed using the Freesurfer analysis package^43^. The dSPM maps for each participant were averaged across conditions and over time, yielding a static map of mean task-induced activity across cortex that was independent of trial type. The anatomical parcellation and averaged dSPM maps were then loaded on each participant’s cortical model simultaneously, and local maxima in the dSPM maps within a priori anatomical regions of interest were defined as ROIs. Consistent with previous studies examining the role of OFC in object recognition^24, 44, 45^, activation in OFC extended posterior to the automated parcellation; this posterior activity was also included in our functional ROIs. Details about the number of vertices taken into consideration for each ROI across participants are provided in Supplementary Table 1.

### Analysis of stimulus-evoked responses

For between subjects analyses, using trial-averaged sensor data we computed the unsigned dSPM timecourse at each vertex for each participant. We then averaged across vertices falling within each ROI, yielding one mean evoked response for each participant and ROI.

To characterize the timing and amplitude of the evoked response in each region, 3 statistics were extracted from each of these waveforms. Onset latency was defined as the latency that satisfied 2 criteria: first, the amplitude at this time point was greater than 2.5 times the standard deviation of the baseline (defined as the 200 ms period prior to stimulus onset), and second, the mean amplitude of the signal over the next 50 ms also satisfied this criterion. Peak amplitude was defined as the maximum amplitude of the signal. Finally, peak latency refers to the latency of the time point in the identified sample with the highest amplitude. These statistics were identified within a temporal window of interest ranging from 50 ms to 300 ms after stimulus onset. This range was chosen because object recognition is thought to occur within 200 ms of stimulus onset^46, 47^. For this between-subjects analysis, we analyzed each of these three evoked statistics using a repeated-measures ANOVA with factors affect condition (positive or negative), ROI, and hemisphere (left or right).

Within subjects, we did not average data across trials. Rather, we extracted our three evoked statistics (onset latency, peak amplitude, and peak latency) from unsigned current estimates based on single-trial data. Data were still averaged across vertices within each subject-specific ROI. These single-trial statistics were analyzed with linear mixed effects models in R using the packages *lme4* ^48^ and *languageR* ^49^. Statistics extracted from evoked waveforms were excluded from analysis if no points in the time series met the criteria for ‘peak onset’, indicating low signal to noise ratio on that trial. Thus, we used linear mixed effects models rather than ANOVA to accommodate missing values. Model parameters included valence (the subject’s most recent 0–10 pleasantness rating on the affect grid), arousal (the most recent 0–10 affect grid rating), image characteristics (color-defined, luminance-defined, or both; see description of stimuli above), image duration, trial number, block number, and induction group (negative or positive) as fixed effects and a random intercept effect due to subject. Significance of fixed effects parameters was assessed using Satterthwaite’s approximation with a significance threshold of p = 0.05. To assess the validity of the mixed effects analyses, likelihood ratio tests were carried out comparing the models with fixed effects to the null models with random effects only. For all significant parameters reported, the parent model performed significantly better than the null model. The number of trials ultimately analyzed in these mixed effects analyses for each ROI is provided in Supplementary Table 2.

### Mediation analysis

A relationship between two variables X and Y is said to be *mediated* by a third variable, M, if X causes M, which, in turn, causes Y. We suspected that the observed effect of valence on response time was mediated by the nature of the stimulus evoked response in different brain regions (i.e., the latency and amplitude of this response). Therefore, we executed a follow up analysis using a series of regressions to test for mediation^50^. For each ROI and evoked statistic of interest, we calculated (1) the beta weight corresponding to the influence of valence on the evoked statistic while controlling for random effects due to subject and (2) the beta weight corresponding to the influence of the evoked statistic on response time while controlling for valence and random effects due to subject. If the product of these two weights divided by their respective standard errors (Z_ab_), was greater than that likely to occur by chance (p < 0.05), the evoked statistic for a particular region of interest was said to mediate the relationship between affect and response time. Arousal ratings, image type, image duration, trial number, block number, and affect induction group were also included as model parameters.

### Phase-locking analysis

Neural synchronization is a putative mechanism for increasing the efficacy of communication between brain regions^25, 51^. Phase-locking quantifies the synchronization of oscillatory activity across brain regions, irrespective of its amplitude^52^. Thus, we performed phase-locking analyses to look for evidence of functional interactions between brain regions.

To perform this analysis, trial-by-trial signed current estimates from each region of interest were filtered with a continuous Morlet wavelet transform (width = 5). Next, we extracted the phase of the signal at each frequency and time point of interest. We then computed our principle statistic of interest, the phase-locking value (PLV), using the following formula:

1 $$PLV(t,f)=\frac{1}{N}|\sum _{n=1}^{N}\exp (j{\varphi }_{1}(t,f,n)-{\varphi }_{2}(t,f,n)|$$

Here, t is time point, f is frequency, n is the trial number, and *ϕ*(*t*, *f*, *n*) is the phase of the MEG waveforms at a given time, frequency, and trial for a pair of ROIs. The PLV measures the consistency across trials of the relative phases of the two signals. The statistic ranges between 0 (no consistent phase difference) to 1 (completely consistent phase difference).

To determine if affect influenced PLV values, we used a nonparametric method developed by Maris and Oostenveld^53^. First, we identified all time-frequency points that showed a p < 0.05 (t-test) uncorrected difference between the positive and negative affect groups (between subjects) or between a median split of trials based on self-reported valence (within subjects – one subject excluded from this particular analysis due to insufficient variation in valence to perform a median split). Next, the mass of the largest cluster (the sum of the t statistics in the largest cluster determined by adjacency in time and frequency) in the time frequency space was found. The sets of PLV values in the given conditions A & B were randomly shuffled between conditions, and the maximal (unsigned) cluster mass was found for each of the random partitions of the data. This procedure was repeated 1,000 times to generate a null distribution of cluster mass values. A cluster in the original contrast was deemed significant if it was in the 95^th^ percentile or greater of null cluster mass values. Because the maximum cluster mass was determined from the entire time-frequency space of interest, this method inherently corrects for multiple comparisons in this space.

### Partial Directed Coherence

We next employed Partial Directed Coherence^54, 55^ (PDC) to assess the directionality of interactions between regions. As with our phase-locking analysis, we began by extracting the signed current estimates from each ROI (averaged across vertices within each ROI) around stimulus onset (−500 to 500 ms). For each ROI pair of interest, we constructed a multivariate autoregressive (MVAR) model for every trial. This model attempted to predict the timeseries of each ROI in the pair based on two factors: the history of the timeseries being predicted and the history of the paired ROI. The MVAR model was fit using the Nutell-Strand algorithm and the model order was chosen using the Akaike information criterion. The coefficients of the autoregressive models were used to compute the PDC in each direction between the two regions of interest. Briefly, the more the history of region B predicts activity in region A, the greater the inferred information flow from region B to A (and vice versa).

More precisely, consider two time series, ${X}_{t}^{1}$ and ${X}_{t}^{2}$, extracted from two different ROIs on a single trial. We constructed an autoregressive model of the form:

2 $$[\begin{matrix}{X}_{t}^{1}\\ {X}_{t}^{2}\end{matrix}]=\sum _{k=1}^{p}[\begin{matrix}{a}^{11}(k) & {a}^{12}(k)\\ {a}^{21}(k) & {a}^{22}(k)\end{matrix}][\begin{matrix}{X}_{t-k}^{1}\\ {X}_{t-k}^{2}\end{matrix}]+\,[\begin{matrix}{\varepsilon }_{t}^{1}\\ {\varepsilon }_{t}^{2}\end{matrix}]$$

where *a* ^*ij*^(*k*) are the autoregressive coefficients and *p* is the model order. Define

3 $${a}^{kl}(\lambda )=\,{\delta }_{kl}-\sum _{s=1}^{p}{a}^{kl}(s){e}^{-\lambda s\sqrt{-1}}$$

where *δ*_*kl*_ = 1 if *k* = 1 and *δ*_*kl*_ = 0 otherwise. The PDC from ${X}_{t}^{j}$ to ${X}_{t}^{i}$ at frequency *λ* is

4 $$PD{C}_{j\to i}(\lambda )=\,\frac{|{a}^{ij}(\lambda )|}{\sqrt{{\sum }_{k=1}^{2}{|{a}^{kj}(\lambda )|}^{2}}}$$

Finally, we integrate PDC values from 0 to 100 Hz. PDC is a granger causality measure in the frequency domain; by computing the PDC and integrating from 0 to 100 Hz, we are able to avoid picking up on spurious evidence of information flow at frequencies near 120 Hz introduced by our lowpass filter that would have contaminated a traditional granger analysis. This integrated PDC value is proportional to the information transfer from region j to region *i* ^55^.

This integrated PDC was computed for all subjects and trials. To asses the presence of information flow in each direction for a given ROI pair (regardless of valence), we calculated the mean PDC across trials for each subject, then calculated the grand mean across all subjects. We then generated a null distribution of grand mean PDCs by repeating this process 1000 times on phase-randomized surrogates of the input time series. The grand mean PDC for a given direction of information flow was deemed significant if values of an equal or greater magnitude were unlikely to occur by chance (p < 0.05). We determined the influence of valence on PDC in a similar manner to our phase-locking analysis: we used t-tests to compare PDC scores between the negative and positive affect groups (between subjects) or between a median split of trials based on self-reported valence (within subjects – one subject again excluded from this particular analysis due to insufficient variation in valence to perform a median split).

## Electronic supplementary material

Supplementary Information
