# Supplementary material for: Internal valence modulates the speed of object recognition
Source: Sci Rep. 2017 Mar 23;7:361. doi: 10.1038/s41598-017-00385-4 (PMC5428282; doi:10.1038/s41598-017-00385-4)
Supplement: Supplementary file 1 — Supplementary Information [file 41598_2017_385_MOESM1_ESM.pdf]

Supplementary Information

Internal valence modulates the speed of object recognition

Matthew F. Panichello[1*], Kestutis Kveraga[2], Maximilien Chaumon[3], Moshe Bar[2,4], and Lisa Feldman Barrett[2,5*]

1. Princeton Neuroscience Institute, Princeton, NJ, USA
2. Athinoula A. Martinos Center for Biomedical Imaging, Massachusetts General Hospital & Harvard Medical School, Charlestown, MA, USA
3. Berlin School of Mind and Brain, Humboldt-Universität zu Berlin, Berlin, Germany
4. Gonda Multidisciplinary Brain Research Center, Bar-Ilan University, Ramat-Gan, Israel
5. Interdisciplinary Affective Science Laboratory, Department of Psychology, Northeastern University, Boston, MA 02115, USA

	Vertices in each region of interest													
**Region**	**ERC**		**Fusiform**		**Insula**		**IPL**		**LOC**		**V1**		**OFC**	
**Hemisphere**	left	right	left	right	left	right	left	right	left	right	left	right	left	right
Negative1	402	714	1667	3145	1336	3068	921	782	2812	978	1228	1309	393	638
Negative2	581	582	2095	1194	684	1541	2295	3058	2218	3393	2827	1531	760	1404
Negative3	1303	580	1686	1326	1050	2787	1679	1281	1675	1474	1652	1276	1164	792
Negative4	816	572	1062	2036	1123	1214	779	765	2377	1340	1301	815	724	750
Negative5	774	794	923	412	1096	2923	1865	2065	1911	2856	1448	1765	355	709
Negative6	663	899	1012	1514	1079	3405	696	1463	1053	1870	1755	2602	681	1094
Negative7	562	747	583	662	1053	2664	586	4110	1025	1368	2112	1698	303	981
Negative8	760	592	746	1032	1627	2018	1923	2251	2377	1729	1579	1722	779	925
Negative9	1079	671	826	2502	1738	892	1236	910	1964	2317	3003	2244	255	439
Negative10	516	494	1817	1417	1422	1471	904	1863	2008	2536	1573	1468	708	963
**Neg Mean**	746	665	1242	1524	1221	2198	1288	1855	1942	1986	1848	1643	612	870
**Neg SD**	272	124	525	832	312	880	605	1077	570	768	615	503	282	268
Positive1	786	593	1078	1731	1112	1271	1019	426	1666	2331	1007	1161	619	680
Positive2	817	679	921	931	1754	1732	517	1907	869	1226	1700	1012	821	552
Positive3	467	368	1618	1582	2213	2336	1983	1190	2291	1321	1355	1110	503	1022
Positive4	887	872	961	2063	1230	1390	2850	2264	1116	772	1284	1651	413	1124
Positive5	828	769	1664	1654	3044	1437	2311	2650	2236	3505	690	1215	450	753
Positive6	677	680	961	1314	974	2843	819	1410	1414	1934	714	953	451	877
Positive7	620	522	325	1630	635	1446	1620	671	803	924	1227	1650	267	259
Positive8	443	530	817	645	733	1526	1574	1284	3001	852	1017	2401	571	811
Positive9	709	415	1146	1107	2481	1119	2090	1317	1991	1990	1059	1343	430	847
Positive10	515	519	2152	1251	845	2100	797	2948	1349	3276	1730	3152	466	1046
**Pos Mean**	675	595	1164	1391	1502	1720	1558	1607	1674	1813	1178	1565	499	797
**Pos SD**	159	157	517	422	829	543	760	822	703	983	357	704	147	257

Supplementary Table 1. Vertices in each region of interest across subjects.

Final trial counts used in mixed models regression analyses

region	left hemisphere	right hemisphere
ERC	2,528	2,467
Fusiform	3,146	3,448
Insula	2,643	2,917
IPL	2,768	3,224
LOC	4,062	4,637
V1	4,582	5,011
OFC	2,904	2,941

Supplementary Table 2. Final number of trials ultimately analyzed for mixed models regression analyses. We presented each subject with 500 trials (10,000 trials total). 7,331 trials remained after removing incorrect trials and trials with oculomotor and muscle artifacts. This population of trials was used for computing mean evoked responses for each subject, and for functional connectivity analyses. For our analysis of trial-by-trial responses using mixed models regression, we also excluded trials on which we could not detect the onset of an evoked response, yielding the values listed above.

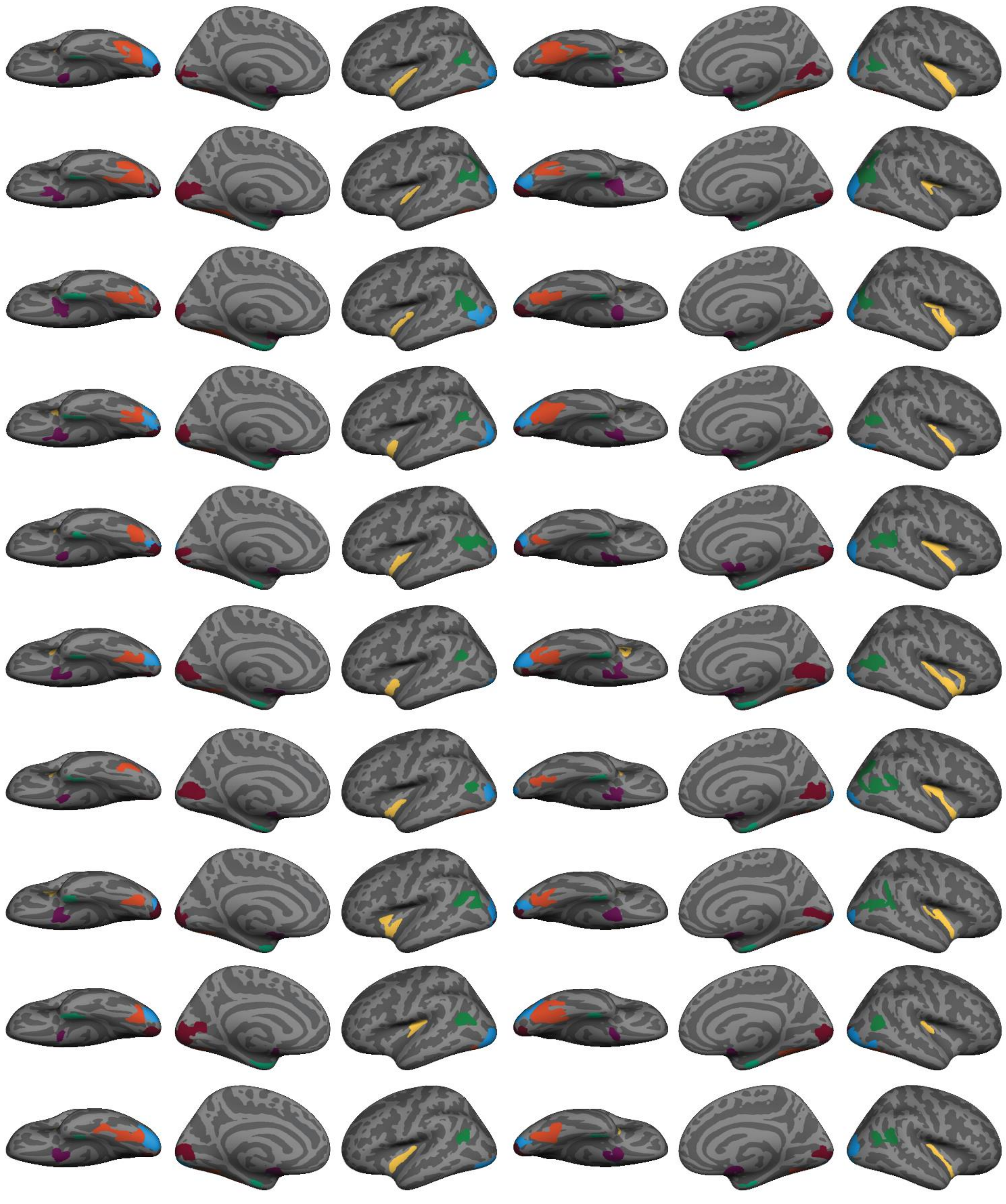

Supplementary Figure 1. Subject-specific ROIs (negative affect group subjects). Each row shows the functionally-defined ROIs for one subject. Maroon = V1, blue = lateral occipital complex, orange = fusiform gyrus, teal = entorhinal cortex, yellow = Insula, green = inferior parietal lobule, purple = OFC.

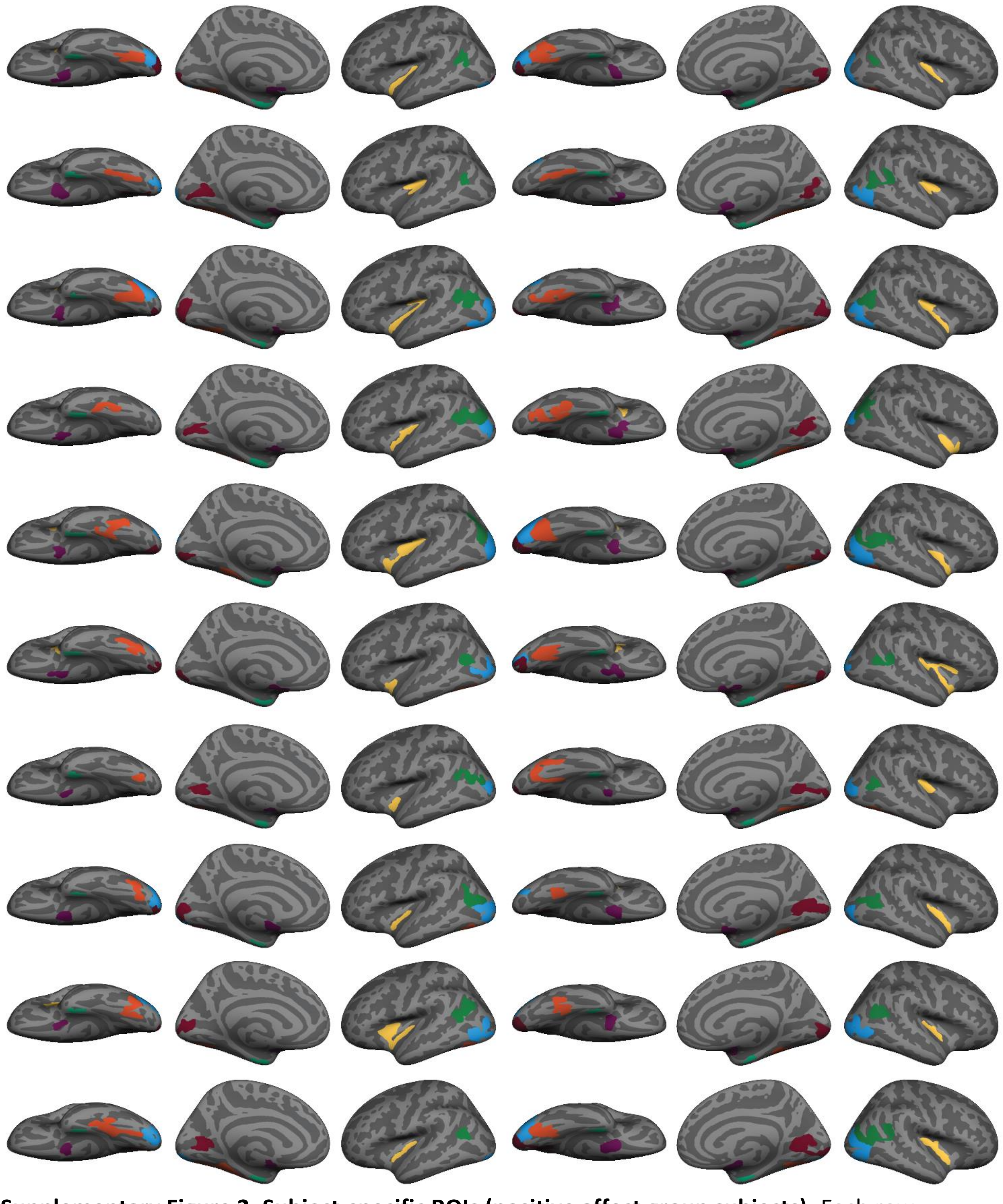

Supplementary Figure 2. Subject-specific ROIs (positive affect group subjects). Each row shows the functionally-defined ROIs for one subject. Maroon = V1, blue = lateral occipital complex, orange = fusiform gyrus, teal = entorhinal cortex, yellow = Insula, green = inferior parietal lobule, purple = OFC.

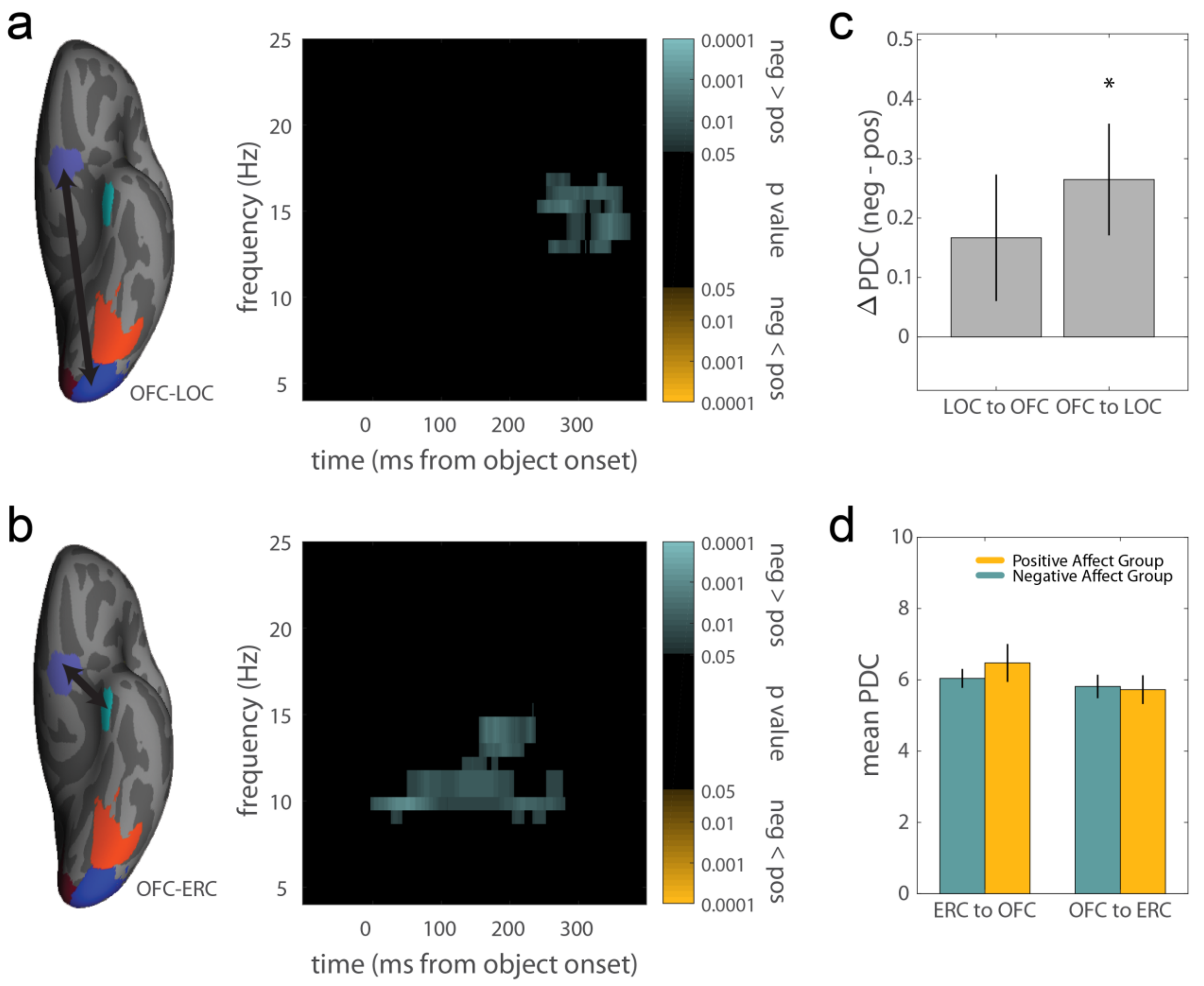

Supplementary Figure 3. OFC functional connectivity varies with valence. a) Within subjects, on trials during which individuals report more negative valence, we observe a late β-band increase in synchrony between left OFC and LOC ($p = 0.059$ cluster-corrected). b) Between affect groups, we observe greater α- and low β-band phase-locking between left OFC and ERC ($p = 0.049$). c) Difference in partial directed coherence between OFC and LOC on negative relative to positive trials (median split). d) Partial directed coherence between OFC and ERC for the negative and positive affect groups.
